# Functional neurological disorder in Europe: regional differences in education and health policy

**DOI:** 10.1111/ene.16350

**Published:** 2024-08-15

**Authors:** Tereza Serranová, Ilaria Di Vico, Michele Tinazzi, Selma Aybek, Ervina Bilic, Stefanie Binzer, Erlend Bøen, Arnout Bruggeman, Christo Bratanov, Veronica Raquel Alheia Cabreira, Dawn Golder, Anna Dunalska, Cristian Falup‐Pecurariu, Beatrice Garcin, Jeannette Gelauff, Aoife Laffan, Simon Podnar, Isabel Pareés, Tom Plender, Stoyan Popkirov, Volodymyr Romanenko, Petra Schwingenschuh, Yury Seliverstov, Carl Sjöström, Matej Škorvánek, Maria Stamelou, Donatas Zailskas, Mark J. Edwards, Jon Stone

**Affiliations:** ^1^ Department of Neurology and Center of Clinical Neuroscience Charles University, 1st Faculty of Medicine and General University Hospital in Prague Prague Czechia; ^2^ Department of Neurosciences, Biomedicine and Movement Sciences University of Verona Verona Italy; ^3^ Faculté des Sciences et de Médecine Université de Fribourg Fribourg Switzerland; ^4^ Department of Neurology Clinical Hospital Centre Zagreb Zagreb Croatia; ^5^ Department of Neurology Kolding Hospital Kolding Denmark; ^6^ Psychosomatic and CL Psychiatry, Division of Mental Health and Addiction Oslo University Hospital Oslo Norway; ^7^ Department of Neurology Ghent University Hospital Ghent Belgium; ^8^ Neurology Department CHU Grenoble Alpes Grenoble France; ^9^ Department of Neurology Geneva University Hospital Geneva Switzerland; ^10^ Neurology Department Centro Hospitalar Universitario de Sao Joao Porto Portugal; ^11^ Department of Clinical Brain Sciences The University of Edinburgh, Royal Infirmary of Edinburgh Edinburgh UK; ^12^ FND Hope UK Banbury UK; ^13^ Psychiatric Clinic of the Faculty of Health Sciences Medical University of Warsaw Warsaw Poland; ^14^ Faculty of Medicine Brasov Transilvania University of Brașov Brașov Romania; ^15^ Department of Neurology Avicenne Hospital, AP‐HP Bobigny France; ^16^ Department of Neurology Vrije Universiteit Amsterdam Amsterdam The Netherlands; ^17^ Department of Neurology St. James's Hospital Dublin Ireland; ^18^ Division of Neurology, Institute of Clinical Neurophysiology University Medical Centre Ljubljana Ljubljana Slovenia; ^19^ Movement Disorders Program, Neurology Department Hospital Ruber Internacional Madrid Spain; ^20^ Movement Disorders Unit, Neurology Department Hospital Universitario Ramón y Cajal Madrid Spain; ^21^ FND Action Chatham UK; ^22^ Department of Neurology, University Hospital Knappschaftskrankenhaus Bochum Ruhr University Bochum Bochum Germany; ^23^ Ukrainian Medical Academy Kyiv Ukraine; ^24^ Department of Neurology Medical University of Graz Graz Austria; ^25^ Department of Neurology Ulm University Ulm Germany; ^26^ Primary Care West Gästrikland Sandviken Sweden; ^27^ Department of Neurology Pavol Jozef Šafárik University Košice Slovakia; ^28^ Department of Neurology L. Pasteur University Hospital Košice Slovakia; ^29^ Parkinson's Disease and Movement Disorders DepartmentHygeia Hospital Athens Greece; ^30^ Department of Neurology and Neurosurgery, Institute of Clinical Medicine, Faculty of Medicine Vilnius University Vilnius Lithuania; ^31^ Institute of Psychiatry, Psychology and NeuroscienceKing's College London London UK

**Keywords:** disability benefits, education curricula, functional neurological disorder, healthcare, patient‐led organization

## Abstract

**Background:**

Functional neurological disorder (FND) is a common cause of neurological disability. Despite recent advances in pathophysiological understanding and treatments, application of this knowledge to clinical practice is variable and limited.

**Objective:**

Our aim was to provide an expert overview of the state of affairs of FND practice across Europe, focusing on education and training, access to specialized care, reimbursement and disability policies, and academic and patient‐led representation of people with FND.

**Methods:**

We conducted a survey across Europe, featuring one expert per country. We asked experts to compare training and services for people with FND to those provided to people with multiple sclerosis (MS).

**Results:**

Responses from 25 countries revealed that only five included FND as a mandatory part of neurological training, while teaching about MS was uniformly included. FND was part of final neurology examinations in 3/17 countries, unlike MS that was included in all 17. Seventeen countries reported neurologists with an interest in FND but the estimated mean ratio of FND‐interested neurologists to MS neurologists was 1:20. FND coding varied, with psychiatric coding for FND impacting treatment access and disability benefits in the majority of countries. Twenty countries reported services refusing to see FND patients. Eight countries reported an FND special interest group or network; 11 reported patient‐led organizations.

**Conclusions:**

FND is largely a marginal topic within European neurology training and there is limited access to specialized care and disability benefits for people with FND across Europe. We discuss how this issue can be addressed at an academic, healthcare and patient organization level.

## INTRODUCTION

Functional neurological disorder (FND) is a common cause of neurological disability and significant healthcare‐related expenditure, comparable to those of other chronic neurological disorders with similar symptoms [1, 2, 3].

FND has heterogeneous presentations including motor and sensory symptoms, seizures, cognitive symptoms and dizziness, that are characterized by the presence of one or more patterns of deficits consistent predominantly with dysfunction of the nervous system and variability (inconsistency) in performance within and between tasks [1].

Over the last decade, advances in FND research have led to a novel conceptualization of the disorder, new diagnostic principles and new treatment pathways [4]. FND is increasingly acknowledged as a complex condition requiring an interdisciplinary (neurology/psychiatry/therapies) approach. The modern approach to FND recognizes the key role of neurologists not only in diagnosing FND but also in the initial management [5]. Importantly, the diagnosis of FND should be based on the presence of positive signs, many of them phenotype‐specific [6, 7]. Promising evidence has accumulated for the efficacy of physiotherapy, psychotherapy or both in the management of FND, for most patients [8, 9]. However, despite these advances and that FND is a potentially treatable condition, there seem to be numerous barriers for people with FND to access early diagnosis and adequate treatment [10].

Several national and international surveys of neurologists from different countries regarding their knowledge and current practice in FND have confirmed that they still lack confidence in establishing the diagnosis and treating patients with FND [11, 12, 13, 14, 15]. In a recent survey among French junior specialists, nearly half of them reported no instruction on FND; only a small fraction was familiar with the Hoover's sign, and the majority felt inadequately trained and informed about FND [16]. In the United States, half of the neurology residents surveyed reported no instruction on treatment of functional seizures, 54% received no training on the utilization of interdisciplinary teams and 13% had no training on functional seizures whatsoever [17]. These omissions in training have consequences. Delayed diagnosis and intervention can result in worse prognosis associated with disease chronicity, prolonged patient suffering and unnecessary medical consultations. According to an Italian study, an average diagnostic delay of 6.6 years correlates with high healthcare utilization costs and poor prognosis [18].

Although the diagnosis of FND generally requires neurological expertise, the management is multidisciplinary. Nonetheless, neurologists have much to offer throughout treatment [9, 19, 20]. A recent viewpoint on FND care in Italy and Czechia highlighted multiple unmet needs and barriers to adequate treatment. These include a lack of specialized services, a noticeable gap in the education and knowledge about FND among healthcare professionals along with insufficient involvement of stakeholders in adopting modern diagnostic criteria and allocating adequate funding in both countries [10, 21]. A detailed survey by FND Hope UK (www.fndhope.org.uk), a patient‐led organization in the UK, revealed variable provision of adequate care for people with FND across the UK [22].

Another key challenge lies in the lack of a unified recognition of FND as a diagnostic entity within current classification systems. It is partially categorized under the Neurology section and partially under the Psychiatric one within the International Classification of Disease (ICD), contributing to significant confusion among physicians and patients and hindering the delivery of high‐standard care [23, 24, 25]. In many countries, if a neurologist makes a diagnosis which is categorized within the psychiatric section of the ICD, they will not be able to receive reimbursement for diagnosis and further management. In addition, disability benefits are often more limited if a disorder has a psychiatric categorization compared to a neurological one [10].

We conducted an expert opinion ‘state‐of‐affairs’ survey to assess education and training in neurology, access to care, reimbursement and disability policies, as well as national academic and patient‐led representation for FND in different European countries. To assess the relative unmet need within each country's healthcare systems, we compared differences in education and access to care with those related to multiple sclerosis (MS), which has a similar prevalence to FND [2, 26] and is associated with comparable disability [27].

## METHODS

A small group of FND experts (M.J.E., T.S., J.S., M.T., I.D.V.) met to identify key areas of interest reflecting unmet needs in the FND field from both a clinician's and patient's perspective. This group created a preliminary questionnaire to collect valid information to map those needs. After multiple iterations, the questionnaire underwent independent external expert review (S.A., B.G., I.P.) to assure comprehensiveness. Final changes were made based on the feedback received. We invited one expert in the FND field from 26 European countries, and where not available, we reached out to academic contacts within the field of movement disorders, and junior doctors with a special interest in FND. Representatives of patient‐led organizations FND Hope UK (www.fndhope.org) (D.G.) and FND Action (www.fndaction.org.uk) (T.P.) also contributed to the final article.

Respondents were encouraged to search for as accurate information as possible from national curricula, local neurological societies, and other FND and MS specialists. Unclear responses or discrepancies in initial survey responses were discussed with the respondents. The survey was conducted via email, with a direct link to the questionnaire provided to participants. It consisted of binary‐choice (yes/no) and multiple‐choice questions and allowed for personal comments to clarify responses.

The survey covered four main areas of interest (see Survey Questions in Supplement 1 in Data S1).

### Section I: Postgraduate education for neurologists

This section explored the inclusion of FND teaching in the training curricula for neurologists across European countries. It gathered data on training programme structures, the incorporation of FND and MS teaching, requirements for trainees to attend specific courses, the number of lectures on FND or MS, and the inclusion of FND in final neurologist examinations.

### Section II: Access to care

This section comprised questions addressing the access to care for FND in general and functional movement disorder (FMD) and functional seizures patients specifically. It investigated the availability of specialized clinics, the number of neurologists with a special interest in FND and MS, and the presence of non‐neurology specialist‐run centres. Furthermore, it inquired about a potential refusal of patient admission at particular facilities if they had a diagnosis of FND.

### Section III: Reimbursement policy and disability payments/benefits

This part inquired about the official diagnostic code for FND, its impact on patient access to treatment and disability/employment benefits, variations in payments for FND diagnosis and treatment compared to other neurological conditions, and the recognition of FND in disability benefit lists.

### Section IV: National academic and/or patient‐led representation of FND

This section sought information on the presence of FND study groups within national neurological societies, the existence of poster sessions dedicated to FND at neurological society congresses, and the presence of patient‐led FND organizations.

#### Statistics

Data analysis involved descriptive statistics, including frequencies and percentages, to present the findings. To examine differences between FND and MS dedicated teaching and services, two‐sample tests for equality of proportions and Wilcoxon signed rank tests were employed. To assess correlation between numerical variables (number of education hours and number of specialists), the Spearman correlation analysis was performed. To assess the association between categorical variables (availability of teaching programme and availability of treatment services), the phi coefficient was calculated.

#### Ethical consideration

The study complies with the Declaration of Helsinki's ethical standards. Given that the respondents are experts from different countries providing opinions on topics of public interest and all co‐authored the study, local ethics approval and informed consent were not sought for this study. The information pertains to professional knowledge and experiences in a specific field; no personal or sensitive data that would pose ethical risks were collected in this study.

## RESULTS

### Respondents' characteristics

Twenty‐five of 27 invited clinicians responded to the survey between 11 May 2022 and 11 March 2023 and then reviewed and updated their responses between 15 January and 20 February 2024.

Most respondents (*n* = 23, 92%) were neurologists; one respondent was a specialist in rehabilitation medicine and psychiatry, and one was a trainee in psychiatry involved in FND/FMD research as a postgraduate student who liaised with colleagues in neurology to identify the data.

Regarding their FND‐related work assignment, nine respondents identified themselves as specialists in a specialized neurology service for FND (i.e., including FMD, functional seizures and other subtypes), five in FMD and none limited to functional seizures. Additionally, there were five general movement disorders specialists seeing also patients with FMD, one specialist in neuromuscular disorders, four general neurologists without a special interest in FND and one respondent working in the rehabilitation medicine department with a specialized service for FND patients.

A full list of respondents and the countries they represented is provided in Supplement 2 in Data S1. The survey competition typically consisted of several rounds with clarifications and updates to ensure the accuracy of the data.

### Section I: Postgraduate education for neurologists

**FIGURE 1:**
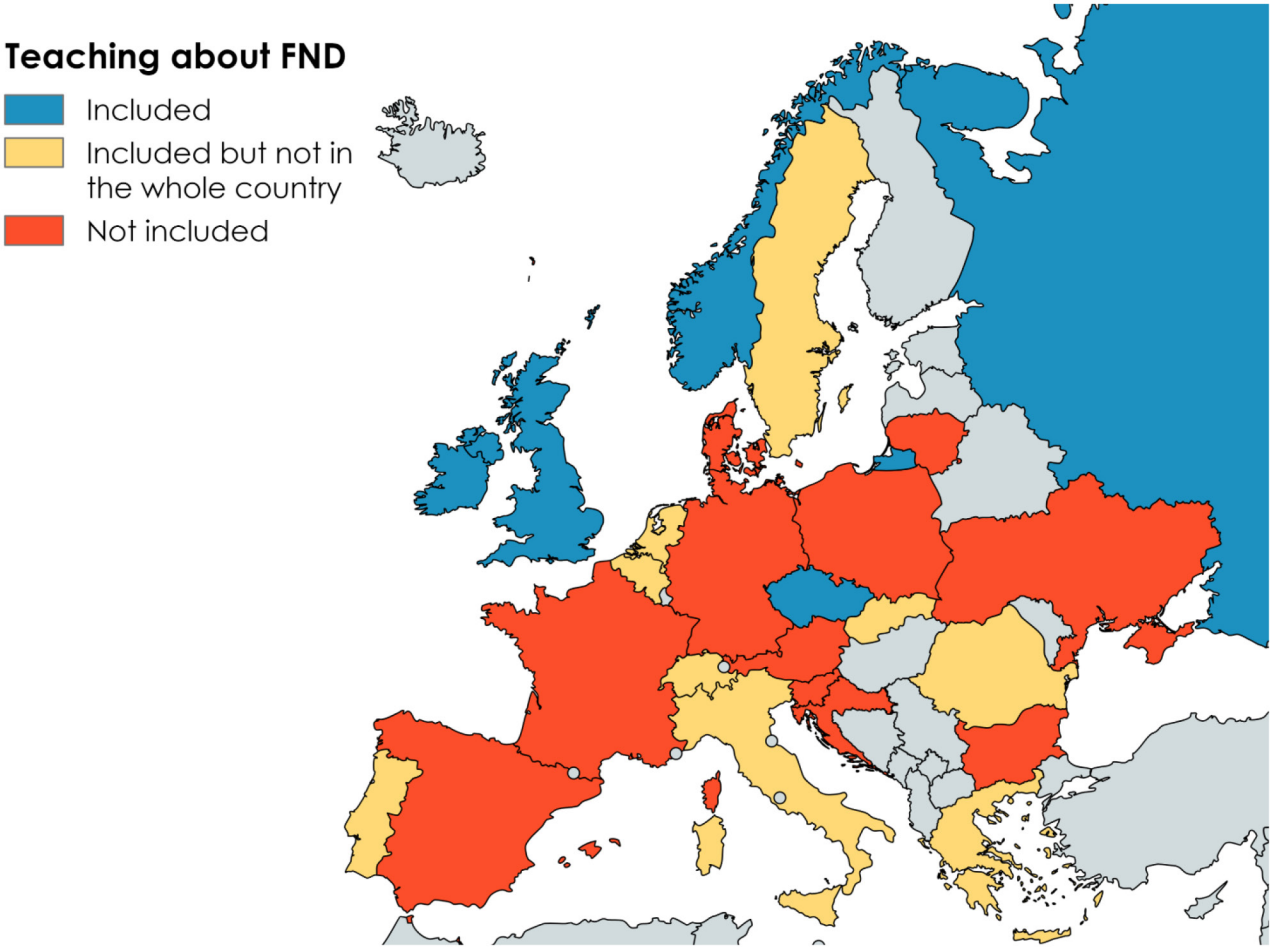
Inclusion of teaching about functional neurological disorder (FND) in the training programmes.

Thirteen of 25 (52%) respondents reported a single national neurology training programme, 36% (9/25) multiple programmes and 12% (3/25) a combination of both single and multiple programmes for neurology training in their countries. Only 20% (5/25) of the curricula included mandatory teaching about FND. In an additional 36% (9/25) of curricula it was included in some, but not all, training programmes or was limited to consideration as a differential diagnosis in one country. Meanwhile, in 33% (11/25) the curriculum contained no training on FND. MS was uniformly included in the training programmes and curricula across all countries (*p* < 0.001) (Figure 1).

While attending a specific MS training programme or course was mandatory in 19 of 22 countries (86%), where this was applicable, an FND training programme was mandatory only in two countries (9%) and in another two countries non‐uniformly (two‐sample test for equality of proportions, *p* < 0.0001).

Respondents from 18 countries provided an estimate of the number of lectures (or hours) on FND and MS per residency period. The mean number of lectures (or hours) on FND per residency was 2.6 (SD 3.5), significantly lower than MS 10.5 (SD 6.5) (Wilcoxon signed‐rank test, *p* < 0.01). In five of the 18 countries, there were no lectures allocated for FND training. The highest ratios of hours/lectures on FMD:MS were reported in the Netherlands (1:1), Austria (3:4) and the UK (1:2), followed by Greece (1:4.5), Russia (1:3.5) and Portugal (1:4). Information about teaching hours/lessons was unknown due to variability or non‐applicable in Bulgaria, Croatia, Germany, Italy, Spain, Sweden and Switzerland.

**FIGURE 2:**
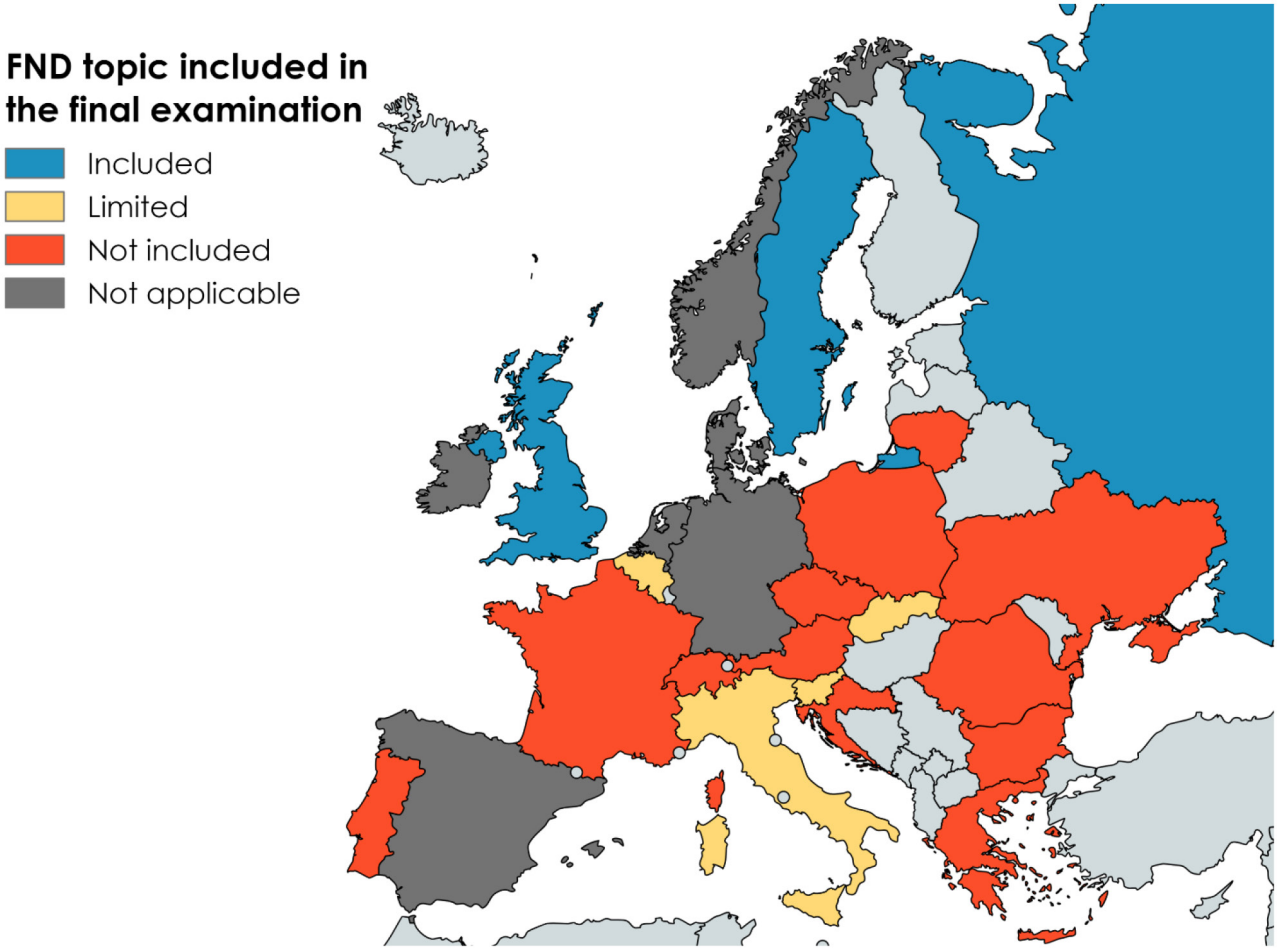
Inclusion of functional neurological disorder (FND) in the final examination for neurology trainees.

In 20 countries a final examination was reported to be part of the neurology curriculum. In 17 of these countries, a yes/no answer regarding the inclusion of FND or MS topic in the final examination curriculum was applicable, with FND included in only 29.4% (3/17) versus 100% (17/17) for MS (two‐sample test for equality of proportions, *p* < 0.001) (Figure 2).

Regarding the comparison of FND training to MS training, 64% (16/25) of respondents reported a very large difference, 28% (7/25) mentioned a large difference, 4% (1/25) stated a moderate difference and 4% (1/25) specified it was non‐applicable.

### Section II: Access to care

The estimated number of specialist neurologists with an interest in FND who run a specialized clinic per number of neurologists was significantly lower than the number of those with an interest in MS with a mean ratio 0.006 (SD 0.006) versus 0.12 (SD 0.16) (Wilcoxon signed‐rank test, *p* < 0.001). Seven respondents reported a complete lack of specialized services in their country.

**FIGURE 3:**
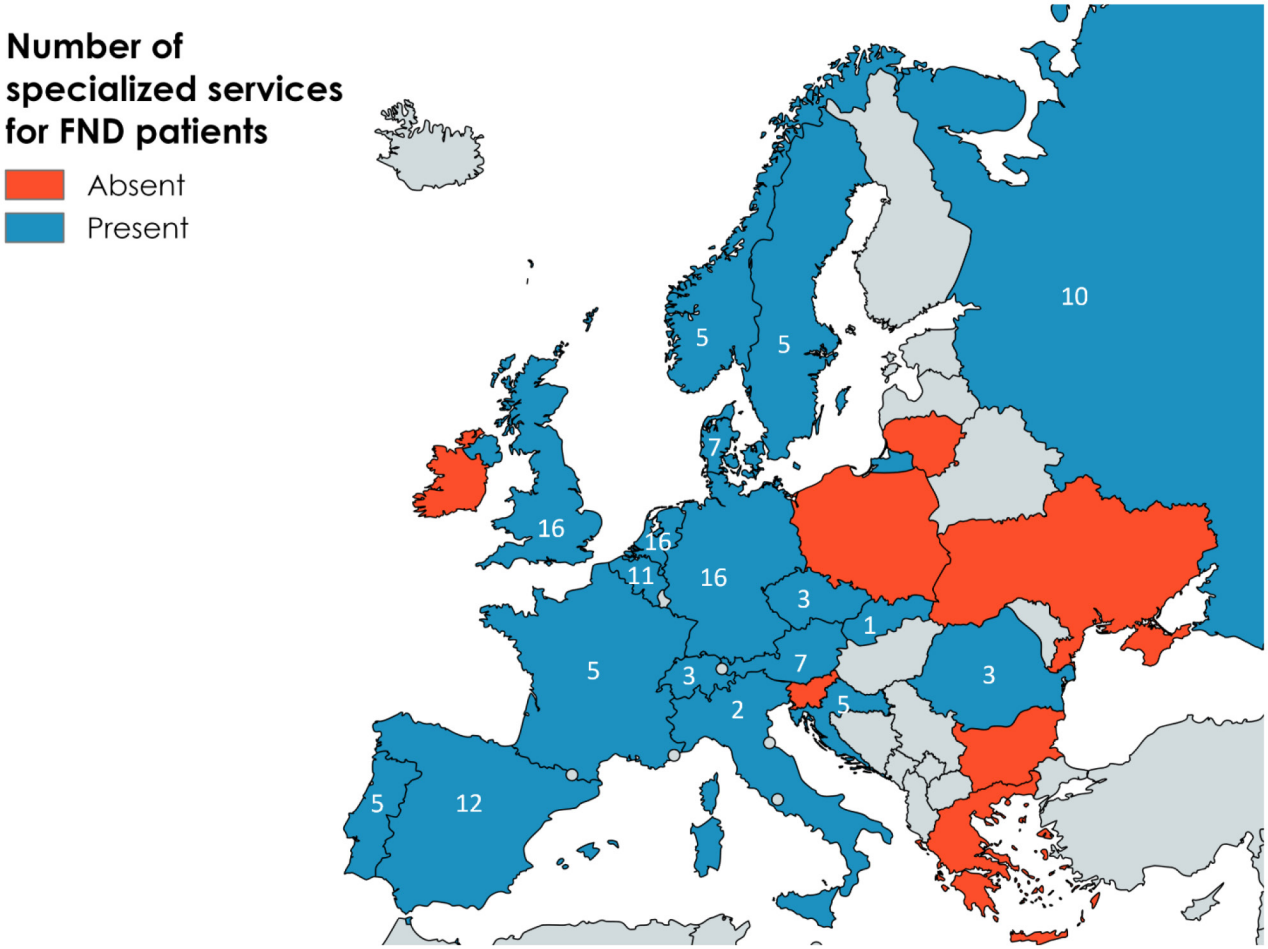
Estimated numbers of specialized centres for people with functional neurological disorder (FND), functional movement disorder and functional seizures in each country.

Results showing the estimated number of specialized clinics in each country are shown in Figure 3.

Seventy‐six specialized services for FND, an additional 22 for functional movement disorder and 36 for functional seizures were reported in all countries.

Twenty‐one respondents (84%) reported that there were other centres in their country without neurologists/specialists with an interest in FND. These centres included psychiatry or psychosomatic medicine services.

Twenty respondents (80%) stated there were centres/services (e.g., rehabilitation inpatient services) that would refuse FND patients, while accepting those with other neurological disorders.

No significant correlation between the number of FND education and numbers of neurologists with an interest in FND (*r*s = 0.28, *p* = 0.25) or MS education hours and the number of MS specialists (*r*s = 0.16, *p* = 0.52) was found. There was a lack of association between the availability of any teaching about FND during residency programmes and availability of any FND specialized services (*rφ =* 0.345, *p* = 0.09).

According to the relative measure of the access to care (i.e., the ratio of specialists with an interest in FND to MS) the highest ratios were reported in Norway (1:4), followed by Germany (1:5), Russia (1:6), Portugal (1:7) and the UK, Ireland and the Netherlands (1:8).

### Section III: Reimbursement policy and disability payments/benefits

A large heterogeneity of terms and official diagnostic codes was reported, with a majority of officially used categories belonging to psychiatric sections of the ICD.

The use of various terms was reported in different countries: “Functional neurological disorder” as the official diagnostic code for FND (*n* = 3, 12%), “Dissociative (conversion) disorder” based on the ICD‐10 coding system (*n* = 8, 32%), “Dissociative neurological symptom disorder” code from ICD‐11 (*n* = 3, 12%) and “Conversion disorder (Functional neurological symptom disorder)” code from DSM‐5 (*n* = 2, 8%). Four respondents (16%) reported that multiple codes, both neurological and psychiatric, were used for FND in their country. Three respondents reported barriers for neurologists in using psychiatric codes and in two countries doctors are not involved in coding.

**FIGURE 4:**
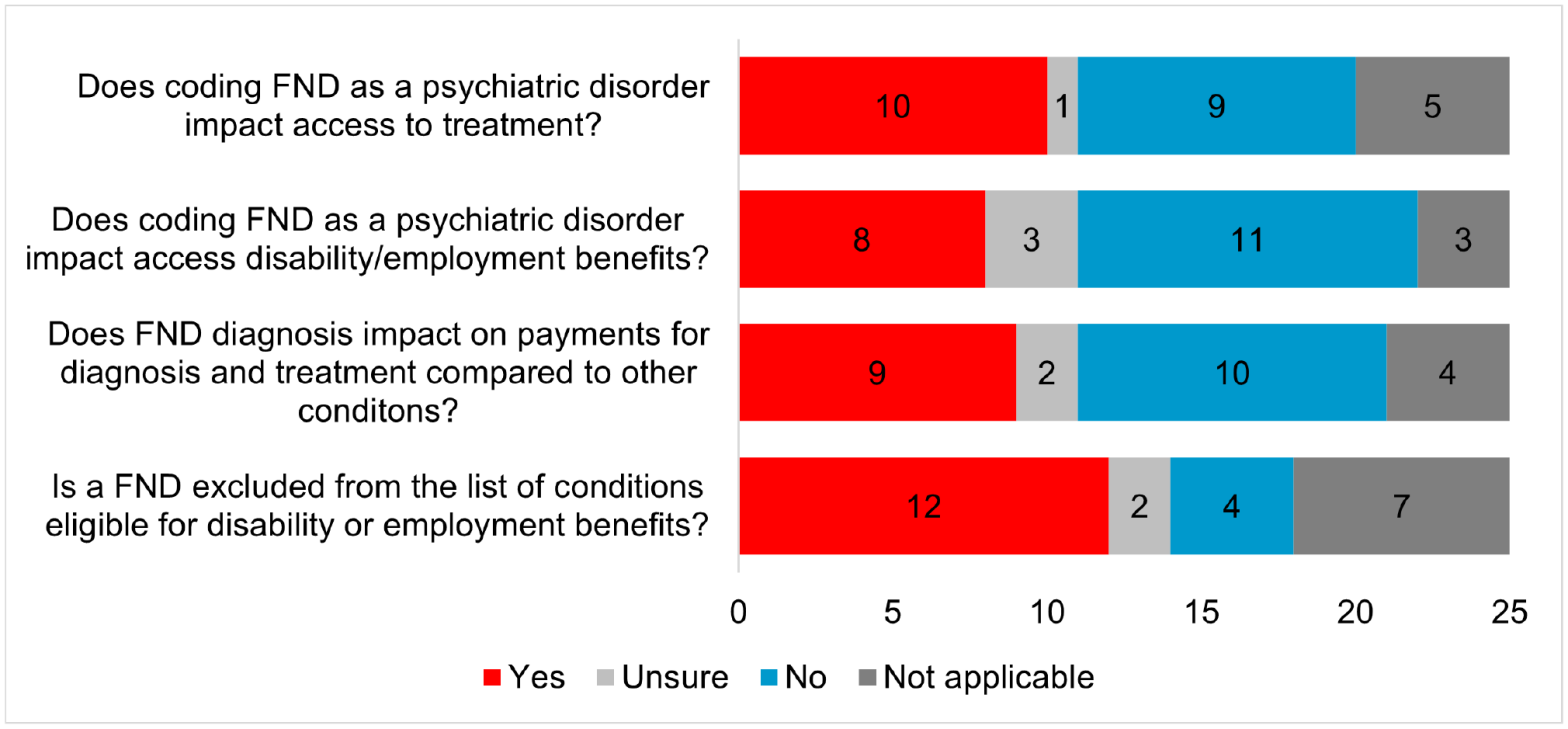
Responses to questions mapping the impact of functional neurological disorder (FND) diagnosis and its coding as a psychiatric disorder to treatment, disability benefits and payments. [Numbers indicate number of countries per answer category.]

Results regarding the impact of a FND diagnosis and its coding as a psychiatric disorder on access to treatment, disability benefits and payments are presented in Figure 4.

Twenty‐three respondents (92%) stated that people with FND had limited access to recognized disability certification and state‐funded disability benefits compared to those with other causes of neurological symptoms; one respondent reported lack of knowledge about disability benefits certification for FND.

### Section IV: National academic and patient‐led representation of FND

The representation of FND within clinical/academic neurology networks was limited. Of the 25 respondents, only five (20.8%) indicated that their country's national neurological society had a study group or section dedicated to FND (Austria, Germany, Italy, the Netherlands, UK). In one country (Sweden), a non‐official, multiprofessional, national network dedicated to FND was reported with approximately 100 members, and three respondents mentioned the existence of a network affiliated or not affiliated with a neurological society (France, Russia).

Twenty‐three respondents (92%) stated that there is no dedicated poster session for FND at the national neurology congress, and two (8%) respondents mentioned a single poster session for all subspecialties.

Of the 25 respondents, 11 (44%) indicated the presence of patient‐led FND organizations or the existence of non‐official patient‐led groups of support such as Facebook groups in their country (Austria, Denmark, France, Germany, Italy, Ireland, the Netherlands, Spain, Sweden, Switzerland, UK), with the UK hosting two internationally relevant organizations.

## DISCUSSION

In this article, we present the findings from a comprehensive expert‐opinion survey from 24 countries, providing an overview of the European FND landscape with a particular focus on FND education and training in neurology, availability of care, and specialized services and policies. Our results reveal a pervasive deficiency in these areas across all surveyed European countries. The scarcity or absence of education and limited access to care becomes even more evident when contrasted with the resources and attention allocated to the field of MS. Additionally, the provided information on prevailing policy frameworks for disability benefits and reimbursement payments offered to FND patients showed significant barriers that people with FND encounter in being recognized as genuinely ill and disabled. Finally, the responses revealed a lack of academic engagement (study groups interested in FND, dedicated sessions in national congresses) and community‐led support (recognized patient‐led organizations) accorded to FND within the European context. The FND field has been growing in the last 10 years from an initial very low base, so it is not surprising that it is taking time for teaching and training to catch up. However, this study allows us to take a snapshot of the progress made and to identify the current gaps.

### Postgraduate education for neurologists

In 2021, the European Academy of Neurology's (EAN) training requirements were updated to include FND as a core topic for the first time [28, 29]. A similar change has followed with the new UK neurology curriculum in 2022 [30]. Despite varied postgraduate education and training across European countries, we found a consistent omission of FND within neurology curricula and training as documented by a significantly lower mean number of lessons on FND compared to MS and a common failure to include FND as a topic in the final examination. Half of the respondents reported no teaching about FND, while teaching about MS was included in all countries with training programmes. Such underrepresentation of FND in postgraduate training serves to increase skill gaps between FND and other neurological diagnoses, likely leading to higher rates of misdiagnosis and perpetuating existing biases in diagnosis and treatment [31].

### Access to care

The results indicate that the current number of FND specialists is low, particularly when compared to a similarly common disorder, namely MS. Several countries reported a complete absence of FND specialists and specialized services. Some 84% of respondents said other specialties, specifically in psychiatry and psychosomatic services, manage FND in their countries. Traditionally, neurologists and psychiatrists used to have distinct perspectives on the management of FND [32, 33]. New recommendations on the role of psychiatrists in the management of FND promote interdisciplinary and multidisciplinary working but this has not filtered through to many health services [34, 35]. For example, 80% of respondents stated that FND patients may be excluded from neurological rehabilitation programmes. There was no significant relationship between education on FND and the accessibility of specialized services for individuals with FND or MS. This suggests that the availability of such services is influenced by a complex interplay of factors, which likely vary across different countries. These factors may include the initiatives of healthcare providers, public and academic representatives, government policies and funding. Such complexity underscores the necessity for a comprehensive understanding and collaboration among all stakeholders to effectively address the challenges faced by those seeking such services.

These findings should prompt stakeholders to develop strategies to address the limited availability of specialized FND care and services across European regions.

### Reimbursement policies and disability benefits

Our survey reveals a lack of standardized FND classification across Europe, with a range of diagnostic codes in use, that ultimately reflects profound confusion and ongoing debates about FND's classification within the medical community. Only 12% of surveyed countries officially use the FND label, while 68% use primarily psychiatric diagnostic codes. This coding seems to have a negative impact on access to care, with a substantial number of respondents noting that a psychiatric label or FND diagnosis influences access to treatment and attribution of disability/employment benefits in their countries. Non‐recognition of FND among the list of the diseases eligible for disability or employment benefits was reported in nearly half of the countries surveyed, while full recognition of FND for benefits was reported only in three. A significant portion of respondents expressed uncertainty regarding this issue, which possibly reflects confusion concerning FND policies and legal rights, and potential inconsistencies in management for FND patients seeking support. A concerning 92% of respondents acknowledged FND patients have limited access to disability certifications and benefits, underscoring the urgent need for better support mechanisms and legal and social protection for these patients.

### National academic and patient‐led representation of FND

Formal representation of FND within national neurological societies (e.g., FND study groups, inclusion of poster sessions dedicated to FND) is notably lacking in the majority of countries. Nevertheless, there are some emerging initiatives, including multidisciplinary networks that could serve as models for fostering collaboration among healthcare professionals and represent important steps in the academic recognition of FND. Patient‐led groups, even Facebook groups that were reported in several countries, have a significant role to play at improving public awareness and lobbying for better health services; however, they are still lacking in most countries.

### Implications and agenda

To address the unmet needs highlighted in our survey, enhancing education emerges as an achievable priority that could improve the situation across the various gaps we have identified. Educational institutions in collaboration with governmental bodies and professional medical organizations or accreditation bodies should do more to integrate FND representation within neurology curricula and training programmes, in keeping with EAN recommendations.

Policymakers and healthcare authorities could address the deficit in access to treatment and benefits. A recent systematic review on the economic costs of FND highlighted unnecessary utilization of healthcare resources and high costs [3]. The authors suggested that interventions – especially those including knowledgeable healthcare professionals for diagnosis and treatment – have the potential to improve patients' health status and reduce costs. While studies on investments in MS have demonstrated increasing investments in disease‐modifying drugs based on evidence and cost‐effectiveness assessments [36, 37], high‐quality data on FND are lacking. Existing evidence and modifiable costs have not been adequately considered [3].

Efforts should also be made in the FND field including changes in classification and the implementation of standardized coding alongside advocacy for the legal rights of FND patients in Europe securing recognized disability and employment benefits according to disability severity. This study identified variations in education and health policies across Europe and pinpointed countries that could serve as models for improving these policies in other regions.

A limitation of an expert‐based survey is that it relies on the opinions and experiences of experts, often in the absence of official sources of reliable data. This means much of the data should be regarded as estimated rather than precise. Due to the large variability in responses, lack of applicability and uncertainty regarding some inquiries it was not possible to compare situations in different countries. Another limitation is that we mostly included experts in FND in general or FMD, with a relative underrepresentation of experts with a specific interest in functional seizures. Our survey examined the provision of education within neurology specialist training, without considering the extent and quality of neurological training in psychiatry, including regarding more neurological aspects of FND. It is crucial to highlight that FND is a neuropsychiatric disorder, which has significant implications for its classification, education, healthcare and policies. This means that defining FND solely within neurological frameworks may lead to limited access to psychiatric care and psychological services. Neglecting either neurological or psychiatric aspects has negative consequences, underscoring the importance of comprehensive understanding and interdisciplinary communication and training, including the development of joint neuropsychiatric curricula. This is not only relevant for FND, but for the large group of patients whose needs span both neurological and psychiatric expertise, and who are therefore often failed by the current schism between the specialties. This might stimulate a wider discussion on the categorization of certain disorders, such as FND, in both the psychiatric and neurological sections of diagnostic classification schemes such as ICD, or even the eventual development of a unified ‘brain disorder’ section.

Despite important gaps that remain to be addressed, several respondents highlighted a rising interest in FND in their countries, with growing efforts to integrate FND into postgraduate neurology curricula along with the emergence of specialized clinics, organizations and societies dedicated to FND. In 2017, the establishment of the Functional Neurological Disorder Society (fndsociety.org) marked a significant milestone. This society has witnessed a growing membership, and currently comprises over 1000 members from various countries (35% Europe as of February 2024). In 2023, the European Academy of Neurology founded a Coordinating Scientific Panel on FND, underscoring a growing understanding of the importance of this area. The increasing involvement of patient‐led organizations across European countries holds promise for raising awareness about this long‐neglected condition. The need for specialized services for people with FND was recently discussed by experts from North America, Europe and Australia who provided a blueprint for developing FND programmes and addressing patient triage, service types, and sustainability challenges within healthcare systems [38]. A recently developed optimal clinical pathway for adults with FND in the UK, supported by National Health System England and the National Neurosciences Advisory Group, could be adapted by future initiatives [39].

## CONCLUSIONS

This study has identified important gaps in FND care and can help in improving awareness of health professionals and educators, increasing national public and governmental attention, and academic dialogue to improve FND care within each participating country. The study outcomes have the potential to facilitate co‐ordinated efforts among European countries to enhance FND care on an international scale.

## AUTHOR CONTRIBUTIONS

**Tereza Serranová:** Conceptualization; methodology; data curation; formal analysis; writing – original draft; investigation; visualization; project administration; writing – review and editing. **Ilaria Di Vico:** Methodology; data curation; writing – original draft; investigation. **Michele Tinazzi:** Conceptualization; methodology; writing – original draft; writing – review and editing; investigation; supervision. **Selma Aybek:** Writing – review and editing; investigation; validation; methodology. **Ervina Bilic:** Investigation; writing – review and editing. **Stefanie Binzer:** Investigation; writing – review and editing. **Erlend Bøen:** Investigation; writing – review and editing. **Arnout Bruggeman:** Investigation; writing – review and editing. **Christo Bratanov:** Investigation; writing – review and editing. **Veronica Raquel Alheia Cabreira:** Investigation; writing – review and editing. **Dawn Golder:** Writing – review and editing; validation. **Anna Dunalska:** Investigation; writing – review and editing. **Cristian Falup‐Pecurariu:** Investigation; writing – review and editing. **Beatrice Garcin:** Investigation; writing – review and editing; validation; methodology. **Jeannette Gelauff:** Investigation; writing – review and editing. **Aoife Laffan:** Investigation; writing – review and editing. **Simon Podnar:** Investigation; writing – review and editing. **Isabel Pareés:** Investigation; validation; writing – review and editing; methodology. **Tom Plender:** Writing – review and editing; validation. **Stoyan Popkirov:** Investigation; writing – review and editing. **Volodymyr Romanenko:** Investigation; writing – review and editing. **Petra Schwingenschuh:** Investigation; writing – review and editing. **Yury Seliverstov:** Investigation; writing – review and editing. **Carl Sjöström:** Investigation; writing – review and editing. **Matej Škorvánek:** Investigation; writing – review and editing. **Maria Stamelou:** Investigation; writing – review and editing. **Donatas Zailskas:** Investigation; writing – review and editing. **Mark J. Edwards:** Conceptualization; methodology; investigation; supervision; writing – original draft; writing – review and editing. **Jon Stone:** Conceptualization; methodology; supervision; investigation; writing – original draft; writing – review and editing.

## FUNDING INFORMATION

The project was supported by the Czech Ministry of Health General University Hospital in Prague project MH CZ‐DRO‐VFN64165 and Charles University: Cooperatio Program in Neuroscience.

## CONFLICT OF INTEREST STATEMENT

The authors declare that there are no conflicts of interest relevant to this work.

## ETHICS STATEMENT

The study complies with the Declaration of Helsinki's ethical standards. Given that the respondents are experts from different countries providing opinions on topics of public interest and all co‐authored the study, local ethics approval and informed consent were not sought for this study. The information pertains to professional knowledge and experiences in a specific field; no personal or sensitive data that would pose ethical risks were collected in this study.

## Supporting information


Data S1.

## Data Availability

The data analyzed in the present study are available upon reasonable request.
