# Supplementary material for: Functional neurological disorder in Europe: regional differences in education and health policy
Source: Eur J Neurol. 2024 Aug 15;31(10):e16350. doi: 10.1111/ene.16350 (PMC11414792; doi:10.1111/ene.16350)
Supplement: Supplementary file 1 — Data S1. [file ENE-31-e16350-s001.docx]

**Supplement 1**

**Survey Questions**

Please answer referring to your own country

*Required.

1. Please provide your name *
2. Which country are you from? *
3. Please provide your job title*
4. Neurologist
5. Psychiatrist
6. Other
7. Are you a specialist in a Specialized Service for *

Mark only one answer.

1. FND
2. Functional Movement Disorders
3. PNES
4. Neurologist without a special interest in FND
5. Other:
6. **Postgraduate education for neurologists**
7. Please specify what is the system of training programs for residents/neurologists in your country.

Mark only one answer.

1. One training program in Neurology for the whole country
2. Multiple training programs (e.g. university based, regional)
3. Other
4. Please provide details about the organization of training programs for residents/neurologists in training in your country. TEXT BOX
5. Does the training program in your country for residents/neurologists in training

include teaching about FND? *

Mark only one answer.

1. Yes
2. Yes, but not in the whole country
3. No
4. Other
5. Is it a requirement that your trainees attend a course or other training program

about FND? *

Mark only one answer.

1. Yes
2. Yes, but not in the whole country
3. No
4. Other
5. Can you specify number of lectures on FND (hours per residency period)

and/or training in FND?

1. Does the training program in your country for residents/neurologists in training

include teaching about MS? *

Mark only one answer.

1. Yes
2. Yes, but not in the whole country
3. No
4. Other
5. Is it a requirement that your trainees attend a course or other training program

about MS? *

Mark only one answer.

1. Yes
2. Yes, but not in the whole country
3. No
4. Other
5. If yes, can you specify number of lectures on MS (hours per residency period)

and/or training in MS?

1. In your country, is there a final examination to become a Neurologist? *

Mark only one answer.

1. Yes
2. No
3. Other
4. If there is a final examination to become a Neurologist, is the topic of FND

included in the final examination curriculum?

Mark only one answer.

1. Yes
2. No
3. Other
4. If there is a final examination to become a Neurologist, is the topic of MS

included in the final examination curriculum?

Mark only one answer.

1. Yes
2. No
3. Other
4. If training/course in FND is present, is it comparable to the level of training in

MS?

Mark only one answer.

1. Very large difference
2. Large difference
3. Moderate difference
4. Minimal difference
5. No difference
6. Other
7. Additional comments on Postgraduate Education and Training – TEXT BOX
8. **Access to care**

This Section is aimed at collecting data on availability of FND specialized Clinics and

access to care for FND patients in your Country

1. Please provide an estimate of the number of neurologists in your country. *
2. Please provide an estimate of the number of neurologists with interest in FND. That is neurologists declare a special interest FND and run a specialized clinic? *
3. Please provide a number of Specialized Clinics for FND
4. Please provide a number of Specialized Clinics for FMD *
5. Please provide a number of Specialized Clinics for PNES *
6. Please provide an estimate for the number of neurologists with interest in Multiple Sclerosis / How many neurologists declare a special interest in Multiple Sclerosis and run a specialized clinic? *
7. Are there other centres without neurologists/ specialists with interest in FND (e.g.

psychiatry or psychosomatic medicine services) in your country? *

Mark only one answer.

1. Yes
2. No
3. Other
4. Please, tell us more about these centres with as much detail as you can – large text box here
5. Are there, in your opinion, centres/services (e.g rehabilitation inpatient services)

that would refuse FND patients while accepting those with other neurological

disorders? *

Mark only one answer.

1. Yes
2. No
3. Other
4. Additional comments on Access to Care - TEXT BOX
5. **Reimbursement policy and Disability payments/benefits**
6. Is there an official diagnostic code for FND in your country that you as a clinician need to follow in your correspondence? *

Check all that apply.

1. Functional neurological disorders
2. Dissociative (conversion) disorder (ICD-10)
3. Dissociative (conversion) disorder (ICD-9)
4. Dissociative Neurological Symptom Disorder (ICD-11)
5. Conversion disorder (Functional neurological symptom disorder) DSM-5
6. Doctors are not involved in coding in my country
7. Others (Please specify below)
8. If FND a is coded as a psychiatric disorder in your country does this affect the patients access to treatment? *

Mark only one answer.

1. Yes
2. No
3. Not applicable
4. Other
5. Additional comments on access to treatment TEXT BOX
6. If FND a is coded as a psychiatric disorder in your country does this affect the access of patients to disability / employment benefits? *

Mark only one answer.

1. Yes
2. No
3. Not applicable
4. Other
5. Additional comments on access of patients to disability / employment benefits – TEXT BOX
6. Are payments for diagnosis and treatment of FND different than those for other causes of neurological symptoms? *

Mark only one answer.

1. Yes
2. No
3. Not applicable
4. Other
5. Additional comments on payments for diagnosis and treatment of FND – TEXT BOX
6. Is FND (or its synonyms) recognized in the list of conditions that have access to disability / employment benefits? *

Mark only one answer.

1. Yes
2. No
3. Not applicable (not a diagnosis based on disability recognition)
4. Other
5. Additional comments on FND recognition – TEXT BOX
6. Do you think people with FND have limited access to recognized disability certification and state funded disability benefits compared to those with other causes of neurological symptoms? *

Mark only one answer.

1. Yes
2. No
3. Other
4. Additional comments on Reimbursement policy and Access to benefits *
5. **National academic and/or government representation of FND**
6. Within the national neurological society in your country, is there a StudyGroup or section interested in FND? *

Mark only one answer.

1. Yes
2. No
3. No – but there is a network not affiliated to a neurological society
4. Other
5. Additional comments on study groups etc. (please, provide weblinks and other resources if available)– TEXT BOX
6. Is there a specific poster session CATEGORY for FND at congresses of the national

neurological society in your country? (i.e. when you are submitting an abstract) or does it have to be submitted in a miscellaneous or other category?

Mark only one answer.

1. Yes
2. No
3. There is not a session for posters
4. Other
5. Are there any patient led FND organizations in your country? *

Mark only one answer.

1. Yes
2. No
3. Other
4. Additional comments on local FND organisations (please, add weblinks or contact details if possible) - TEXT BOX
5. General comments on FND representation *
6. General comments on the survey

**Supplement 2**

**Map of European countries involved in the Survey and list of respondents**


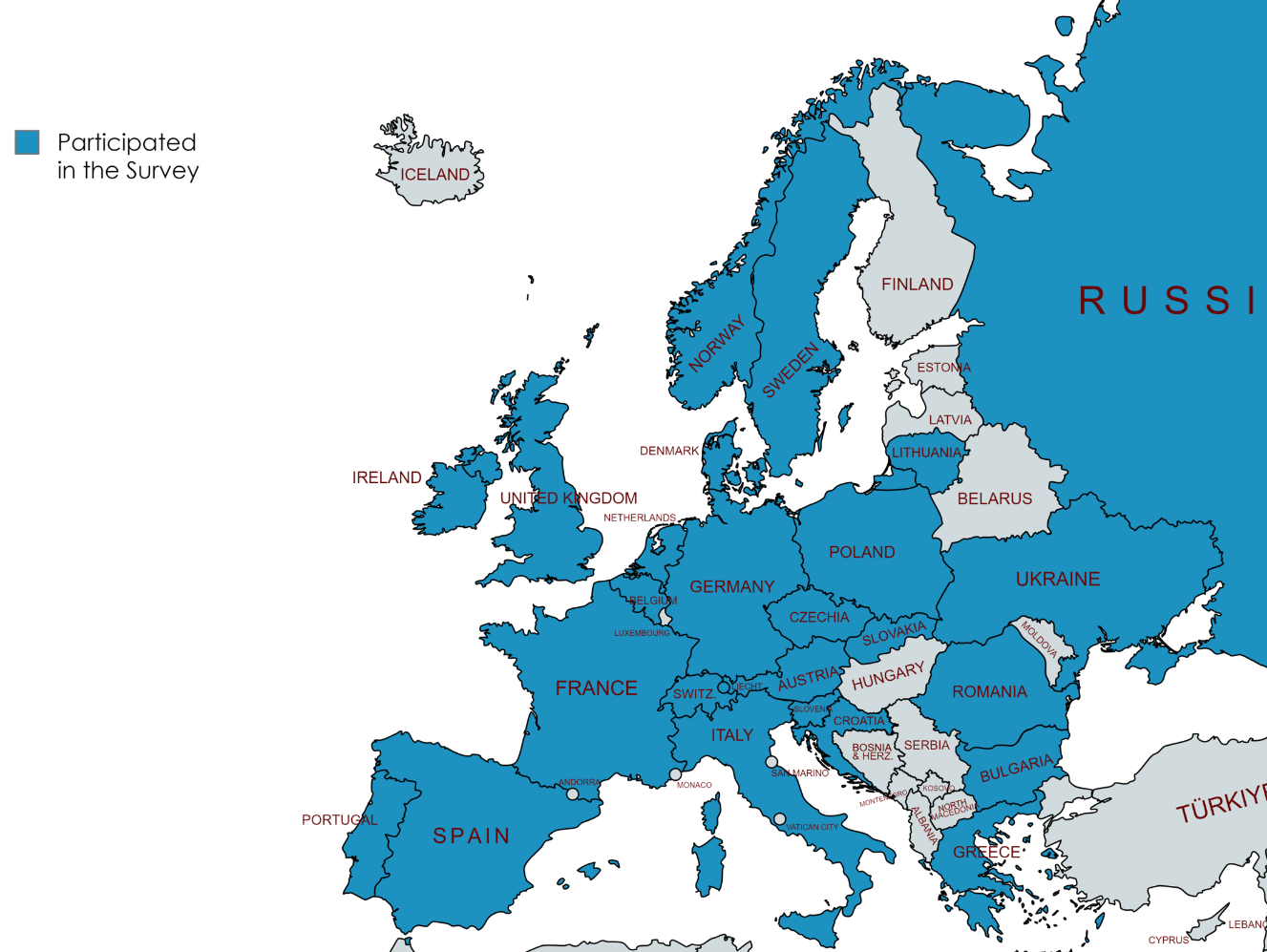


| Petra Schwingenschuh | Austria | neurologist |
| --- | --- | --- |
| Arnout Bruggeman | Belgium | neurologist |
| Christo Bratanov | Bulgaria | neurologist |
| Ervina Bilic | Croatia | neurologist |
| Tereza Serranová | Czechia | neurologist |
| Stefanie Binzer | Denmark | neurologist |
| Beatrice Garcin | France | neurologist |
| Stoyan Popkirov | Germany | neurologist |
| Maria Stamelou | Greece | neurologist |
| Aoife Laffan | Ireland | neurologist |
| Michele Tinazzi | Italy | neurologist |
| Donatas Zailskas | Lithuania | neurologist |
| Erlend Bøen | Norway | neurologist, psychiatist |
| Anna Dunalska | Poland | psychiatry trainee |
| Veronica Raquel Alheia Cabreira | Portugal | neurologist |
| Cristian Falup-Pecurariu | Romania | neurologist |
| Yury Seliverstov | Russia | neurologist |
| Matej Skorvanek | Slovakia | neurologist |
| Simon Podnar | Slovenia | neurologist |
| Isabel Parees | Spain | neurologist |
| Carl Sjöström | Sweden | rehabilitation medicine, psychiatry |
| Selma Aybek | Switzerland | neurologist |
| Jeannette Gelauff | The Netherlands | neurologist |
| Volodymyr Romanenko | Ukraine | neurologist |
| Jon Stone | United Kingdom | neurologist |
